# Review on the conceptual framework of teacher resilience

**DOI:** 10.3389/fpsyg.2023.1179984

**Published:** 2023-07-20

**Authors:** Shen Zhang, Yuzhou Luo

**Affiliations:** ^1^School of Social Sciences, Tsinghua University, Beijing, China; ^2^Guangzhou College of Commerce, Guangzhou, China

**Keywords:** conceptual analysis, correlated variables, effecting factors, affected variables, teacher resilience

## Abstract

Resilience is the ability to bounce back from setbacks and adapt to new circumstances. Resilient teachers can handle these issues. In this case, it’s proposed to interpret the recent decade’s resilience research on teachers. Provide a conceptual framework for teacher resilience factors. The Scopus database was used to collect articles. The titles and abstracts of articles were read one by one. As a result, 22 articles were included in the data analysis. The country where the data were collected, the aims of the study, the education level which the participants working, the sample size, the scale used, and the variables included in the study are marked in the full text. Most studies were effect determination, correlation, or exploratory. Initially, age and gender inequalities among instructors were examined. Postgraduate instructors are more resilient than undergraduates. Psychological factors, workplace variables, and teacher competency and attributes are used to study teacher resilience. Teachers’ resilience negatively impacts depression, stress, anxiety, well-being, and mood. Quality of life and well-being are positively connected. Job crafting, work engagement, and working environment are favorably connected, whereas job burnout and turnover intention are adversely correlated. Resilience was positively connected with emotion regulation, empathy, others’ emotion evaluation, teacher competence, teacher self-efficacy, and self-esteem in teachers. Anger, anxiety, mindfulness, pleasure, social support, fear, and training affect teachers’ resilience. Teachers’ resilience affects stress, depersonalization, personal accomplishment, emotional exhaustion, children’s resilience, job engagement, happiness, well-being, self-care, and success.

## Introduction

Teacher resilience is a crucial topic in the world of education, especially considering the multiple obstacles and pressures that teachers confront every day (Brouskeli et al., 2018; López-Angulo et al., 2022). Resilience refers to an individual’s capacity to overcome adversity, recover from failures, and adapt to changing conditions (Bobek, 2002; Kangas-Dick and O’Shaughnessy, 2020). Resilience in the context of teaching is the capacity of teachers to sustain their effectiveness and well-being despite the numerous demands and constraints of the job (Mansfield et al., 2016).

Day and Gu (2014) take exception to the notion that resilience can be summed up as nothing more than the ability to recover quickly after experiencing difficult or traumatic events. The idea of resilience is dynamic and multidimensional, and it is possible to cultivate it through the interaction of one’s own resources and the resources provided by their environment (Peixoto et al., 2020). To be more precise, it has been discovered that teachers’ levels of resilience are neither natural nor consistent, but rather vary as a direct result of the impacts exerted by the personal, social, and organizational contexts in which they operate (Gu and Day, 2013). In a study conducted by Mansfield et al. (2012), the researchers questioned 200 preservice and early career teachers, “what makes a resilient teacher?” The study’s focus was on the protective characteristics that enhance teacher resilience. The findings highlighted four broad dimensions of protective factors: those specifically related to the profession (such as self-efficacy beliefs and pedagogical competencies), emotional aspects (such as positive emotions and emotional management), social aspects (such as supportive relationships with students and colleagues), and motivational aspects (such as having a sense of purpose in one’s work) (e.g., intrinsic motivation, persistence, expectations, and goals). These findings served as the foundation for the development of a scale that takes into account the multifaceted character of resilience within the context of the teaching profession.

Concerns about high rates of teacher burnout, attrition, and discontent have contributed to an increase in research on teacher resilience in recent years (Gratacós et al., 2021). Studies (Brouskeli et al., 2018; Suryaratri et al., 2020; Diasti, 2021) investigated a variety of elements that contribute to resilience, including personal qualities, social support, coping techniques, and occupational resources. By gaining a deeper knowledge of these elements, educators and policymakers may design interventions and methods to increase teacher well-being, job satisfaction, and student results (Van Wingerden and Poell, 2019; Daniilidou et al., 2020; Cho et al., 2021).

Teacher resilience is an important area of study because teachers who do a good job and stay in the field have a positive effect on their students’ learning. Teacher resilience, along with their knowledge, skills, and other qualities, make up a patchwork of learning support that helps students do better in school. It’s even more important when you think about how teachers are the most important resource for making sure students learn well, especially in an emerging economy with few resources (Ebersöhn, 2014). Teacher resilience sees itself as a concept that bridges the gap between the complicated contexts of practice and the people who work in them. So, this paper looks at the transition from the individual to the school context. It suggests that teacher training should go the other way, from the school context to the person (Gratacós et al., 2021).

Even though it has been defined in different ways, teacher resilience seems to be a mix of personality traits, developmental processes, and skills that teachers have learned (Bobek, 2002; Benders and Jackson, 2012; Ebersöhn, 2014; Tenorio-Vilchez and Sucari, 2021). Resilience is an important part of what keeps new teachers in the job. From a career psychology point of view, teacher resilience is related to work engagement (Van Wingerden and Poell, 2019; Xie, 2021), burnout (Daniilidou et al., 2020; Liu et al., 2021), and job satisfaction (Li and Lv, 2022). It has been linked to a teacher’s ability to help kids be resilient and a desire to leave the teaching profession (Bouillet et al., 2014; Bowles and Arnup, 2016). It has been called a key factor for teachers who work in poor urban areas (Day and Hong, 2016; Suryaratri et al., 2020) and for teachers who work with kids who have special educational needs (Mackenzie, 2012; Abdullah et al., 2019).

The job of a teacher is getting more difficult, demanding, and tiring (Flores, 2020; Park et al., 2020). The motivations behind this study are to further explore the complex nature of teacher resilience and identify the factors that contribute to it. By examining the recent literature, this study aims to create a comprehensive and up-to-date conceptual framework of teacher resilience.

The specific objectives of this study are to:

1. Investigate the variables related to teacher resilience in studies conducted over the last 10 years.
2. Develop a conceptual framework that integrates the findings of these studies and captures the multidimensional nature of teacher resilience.

To address these objectives, the study focuses on research conducted in the last 10 years. This time frame was chosen to capture the most recent trends and developments in the field of teacher resilience, ensuring the conceptual framework is relevant and applicable to current educational contexts. By achieving these objectives, this study aims to provide a better understanding of the factors that contribute to teacher resilience, ultimately informing interventions and strategies to enhance teacher well-being, job satisfaction, and student outcomes.

## Method

It is aimed to construct a mini review on the study teacher’s resilience. The study is based on published articles. The study is based on published articles, and a systematic review procedure following the PRISMA method was employed.

### Data collection process

The Scopus database was used to collect published studies. Scanned using the keyword “teacher resilience.” Articles published in English in the last 10 years were selected. As a result of the restriction, 172 articles were seen. The data from the obtained studies has been downloaded in CSV format. The titles and abstracts of 172 articles were read one by one. It was examined according to the criteria of measuring the resilience level of teachers, using a quantitative measurement tool, and not having a review or meta-analysis study. Since the meta-analysis studies were based on published studies, they were excluded from the scope due to the absence of duplication in the studies. As a result of the preliminary examination, 31 articles were selected. In the next step, their full texts were reviewed to determine whether their work fits the focus of the study. In this review, nine articles that did not meet the criteria, such as studies on pre-service teachers, were excluded from the study. As a result, 22 articles were included in the data analysis.

### Data analysis

The researchers read each of the 22 articles they obtained as a result of the review one by one. The country where the data were collected, the aims of the study, the education level at which the participants worked, the sample size, the scale used, the number of citations, and the variables included in the study are marked in the full text (Table 1).

**Table 1:** Description of studies in teacher resilience.

Id	Authors	Country	Aims	*N*	School level	Spe.	Data collection tool	Cited by
1	Abdullah et al. (2019)	Malaysia	C	<100	S	SE	SECRS^12^	0
2	Ayoobiyan and Rashidi (2021)	Iran	C, E	<100	U	EFL	CDRS^2^	12
3	Baguri et al. (2022)	Malaysia	C	>100	P, S	U	BRS^3^	4
4	Bouillet et al. (2014)	Croatia	C, E	>100	Pre	pre	RS^9^	10
5	Brouskeli et al. (2018)	Greece	Ex, C	>100	S	MG	RS^1^	32
6	Cho et al. (2021)	South Korea	C	>250	P, S, H	MG	BRS^3^	5
7	Daniilidou et al. (2020)	Greece	C, E	>500	P	U	MTRS^4^	10
8	Fernandes et al. (2019)	Portugal	E	<100	P, S	U	RS^7^	26
9	Gan et al. (2022)	China	C, E	>250	U	EFL	CDRS^2^	1
10	Gratacós et al. (2021)	Spain	Ex, C	>100	Pre, P	MG	MTRS^4^	7
11	Khammat (2022)	Iraq	C, E	>250	H	EFL	RS^5^	0
12	Kowitarttawatee and Limphaibool (2022)	Thailand	E	>250	Uni	U	ER^10^	2
13	Li and Lv (2022)	China	C, E	>250	U	EFL	RS^5^	1
14	Liu et al. (2021)	China	C	>500	h	U	CDRS^2^	19
15	Liu et al. (2022)	China	C, M	>500	P, S	MG	MTRS^4^	9
16	López-Angulo et al. (2022)	Chile	Ex, C	>500	U	MG	RS^13^	0
17	Park et al. (2020)	South Korea	C, E	>250	P	Pre	RS^11^	1
18	Pečjak and Pirc (2022)	Slovenia	E	>500	P, S	U	CDRS^2^	0
19	Suryaratri et al. (2020)	Indonesia	E	>100	H	U	CDRS^2^	0
20	Van Wingerden and Poell (2019)	Netherlands	C	>100	P	U	RS^8^	0
21	Xie (2021)	China	C, E	>300	U	EFL	CDRS^2^	12
22	Yirci et al. (2022)	Turkey	E	>250	Pre, P, S, H	U	BRS^3^	2

Aims: C, correlation; E, determining the effect; Ex, exploratory; School level: Pre, preschool; P, primary school; S, secondary school; H, high school; Uni, University; U, unspecified; Specialization: SE, special education; EFL, English as a foreign Language; MG, multiple groups; Pre, preschool teachers; U, unspecified. Superscript numbers provided were used to determine the same data collection tool.

## Findings

When the number of publications is analyzed by years, the increasing number of publications over the years indicates that there is an increasing interest and focus in the field of teacher resilience research. In 2014 and 2018, only one publication was recorded per year. The number of publications increased to four in 2019 and then to three in 2020; this indicates steady growth in interest and research into teacher resilience. With six articles published in 2021, there was a significant increase in the number of publications. In 2022, the uptrend continued with seven posts recorded so far. This highlights the continued interest and commitment of researchers to explore various aspects of teacher resilience, refine methodologies, and examine new contexts and dimensions. In summary, the increasing number of publications from 2014 to 2022 indicates that the importance of teacher resilience research is increasingly recognized. As more studies are conducted, it is important to integrate and synthesize the findings to contribute to a comprehensive understanding of teacher resilience and its implications for education.

Examining the research on teacher resiliency reveals two studies having a single author, while the remaining studies have multiple authors. When the countries researched were categorized, the majority of studies, eleven, were done in East Asia. Thereafter, seven studies were conducted predominantly in Europe. The Middle East (3) and South America (1) are positioned next on the list. When the aims of the conducted studies were categorized, the majority consisted of effect determination (13), correlation (11), and exploratory (3) studies. There was a categorization of sample size. Five of the studies have samples of 500 or more, while eight contain samples between 250 and 500. Although six studies include between 100 and 250 participants, the other three studies have less than 100 people. Examining the categorization of sample groups according to education level reveals that some studies were conducted at a single education level while others were conducted at many levels. Most research was conducted in elementary (8) and secondary (7) schools. In the subsequent ranking, the high school (5) and preschool (4) levels were included. One research was done at the university level, while the level was unspecified in five other investigations. When the specialties of the teachers were analyzed, it was shown that EFL teachers predominantly operated as a unit. In 5 studies, several branch instructors were identified, but in 2 studies, preschool teachers and in 1 study, special education teachers were examined. In nine investigations, there was no explanation about the branches of the professors.

When the scales used to measure the resilience of teachers in the studies were examined, the CDRS scale based on the Connor and Davidson (2003) study was used the most. Later, the Brief Resilience (Smith et al., 2008) and the Multidimensional Teacher Resilience Scale (MTRS) (Mansfield and Wosnitza, 2015) scales were used. Teacher Resilience Questionnaire (Campbell-Sills and Stein, 2007) study was used twice. Other scales were used once.

The article by Brouskeli et al. (2018) has the highest number of citations with 32, indicating that it is a highly influential study in the field of teacher resilience. The high citation count may suggest that the findings or methodology of this study are particularly relevant to other researchers in the field. The articles by Fernandes et al. (2019) and Liu et al. (2021) have also received a significant number of citations, with 26 and 19, respectively, suggesting that these studies have also had a notable impact on the research community. The majority of articles have a citation count of 12 or below, which may indicate that these studies are relatively new or have had a more modest influence in the field. It is important to note that articles published more recently, such as Khammat (2022) and López-Angulo et al. (2022), have not had as much time to accumulate citations, and thus their impact on the field might not be fully reflected in their current citation count. There are several articles with zero citations, such as Abdullah et al. (2019), Suryaratri et al. (2020), and Khammat (2022). These articles may be less influential or might have been published very recently, giving them less time to be cited by other researchers. Overall, the variation in citation counts among these articles highlights the diverse range of influence and impact that these studies have had in the field of teacher resilience. The varying citation numbers also emphasize the importance of considering multiple factors, such as publication date and overall trends in the field, when evaluating the impact of these articles.

### Variables in teacher resilience studies

**Figure 1:**
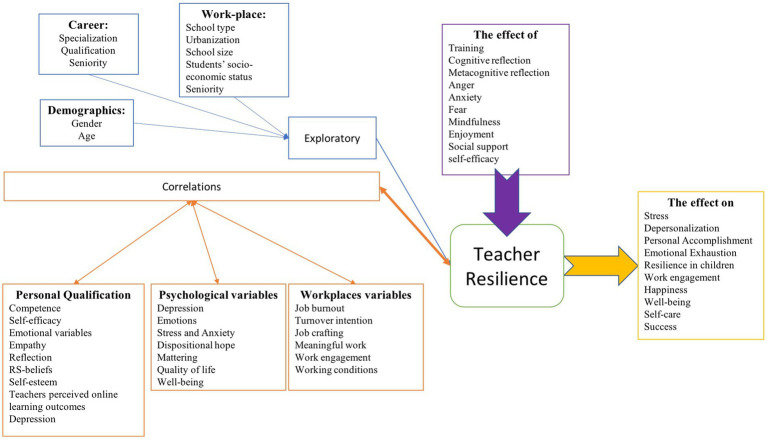
Conceptual map of teacher resilience.

The researchers seek to see if the levels of teacher resilience as evaluated by the teachers altered depending on some variables (Figure 1). To begin, it was investigated whether or not there was any variation among the teachers with regard to demographic factors such as age and gender. In spite of the fact that some studies (Brouskeli et al., 2018; Van Wingerden and Poell, 2019) suggests that the levels of teachers’ resilience do not vary depending on the gender variable, another study (Liu et al., 2022) found that women had higher levels of resilience than men, while the result of the study (López-Angulo et al., 2022) indicated that men had higher levels of resilience than women. According to the age variable, the findings of the study (Liu et al., 2022) indicate that experienced instructors with an age range of 36–45 years have a greater level of resilience than others. There was no difference found between the ages of those who participated in the studies (Brouskeli et al., 2018; Van Wingerden and Poell, 2019).

Also, variables pertaining to teachers’ careers were studied. According to Brouskeli et al. (2018), teachers of the humanities and social sciences are more resilient than those of the exact and natural sciences. Pedagogy in language, communication, and Spanish instructors have more resilience than pedagogy in mathematics and computer Science, according to the finding (López-Angulo et al., 2022). Nevertheless, according to Brouskeli et al. (2018), postgraduate instructors are more resilient than their undergraduate counterparts. Although Brouskeli et al. (2018) indicate that there is no differentiation based on teacher seniority, Liu et al. (2022) indicate that the resilience levels of new instructors (those with 1–3 years of experience) are lower than those of other teachers. In the study (Brouskeli et al., 2018), it was determined that the resilience of teachers did not change according to the variables of school type, school size, and students’ socio-economic status.

In studies that examine if there is a correlation between teacher resilience and specific variables, the variables are categorized under three headings: psychological variables, variables connected to the workplace, and variables related to teachers’ own competence and qualities. According to the findings (Cho et al., 2021), there is a negative correlation between teachers’ resilience and depression, stress, and anxiety. According to the results (Gan et al., 2022), emotion and resilience are negatively correlated. On the other hand, according to Baguri et al. (2022) findings, dispositional hope and mattering are positively correlated with resilience. In addition, there is a favorable association between resilience and quality of life (Abdullah et al., 2019), and well-being (Brouskeli et al., 2018; Cho et al., 2021). Although job burnout and turnover intention (Liu et al., 2021) were correlated negatively with resilience, job crafting, meaningful work, work engagement (Van Wingerden and Poell, 2019), and working conditions (Brouskeli et al., 2018) were correlated positively with resilience. Resilience was shown to be positively associated with emotion regulation (Xie, 2021; Khammat, 2022; Li and Lv, 2022; López-Angulo et al., 2022), use of emotion (López-Angulo et al., 2022), total emotional intelligence (López-Angulo et al., 2022), and self-emotional appraisal (López-Angulo et al., 2022), which may be viewed as instructors’ capacity to regulate emotional states. In this study, a positive correlation was identified between resilience and empathy (Pečjak and Pirc, 2022) and others’ emotion evaluation (López-Angulo et al., 2022), which are connected to the ability to comprehend the emotional states of their colleagues and pupils. There is a positive correlation between resilience and teacher competence (Brouskeli et al., 2018; Liu et al., 2022; Pečjak and Pirc, 2022), teacher self-efficacy (Cho et al., 2021; Gratacós et al., 2021), crisis self-efficacy (Baguri et al., 2022), reflection (Ayoobiyan and Rashidi, 2021), and self-esteem (Baguri et al., 2022) in terms of the teachers’ personal qualities. It has been discovered that there is a positive correlation between instructors’ views about some of their topics (RS-beliefs; Pečjak and Pirc, 2022) and their perceptions of online learning outcomes (Liu et al., 2022) and resilience.

There were two categories that the impact studies fell into. Within the first category are the elements that have an effect on resilience. Anger (Gan et al., 2022), anxiety (Gan et al., 2022), and fear (Yirci et al., 2022) all have a detrimental impact on one’s resilience. On the other hand, beneficial effects are produced by positive emotional states such as Eastern mindfulness (Kowitarttawatee and Limphaibool, 2022), Western mindfulness (Kowitarttawatee and Limphaibool, 2022), and enjoyment (Gan et al., 2022). In addition, the findings of the study (Ayoobiyan and Rashidi, 2021) indicate that mental abilities such as cognitive reflection and metacognitive reflection have a favorable influence on one’s resilience. The resilience of teachers can be increased by training on resilience (Bouillet et al., 2014; Fernandes et al., 2019) and through social support (Park et al., 2020; Suryaratri et al., 2020). According to the findings of the experiment (Daniilidou et al., 2020), the self-efficacy and resilience of instructors are favorably affected. The second group consists of the many factors that are subjected to research about the influence of resilience. For instance, according to the findings (Daniilidou et al., 2020), it has a detrimental impact on both resilience and depersonalization, as well as emotional tiredness and stress. Happiness (Yirci et al., 2022), psychological well-being (Khammat, 2022), personal accomplishment (Daniilidou et al., 2020), self-care (Park et al., 2020), success (Li and Lv, 2022), teachers’ job engagement (Xie, 2021), and resilience in children (Bouillet et al., 2014) are all favorably influenced, though.

## Conclusion and recommendations

The rise in the number of publications between 2014 and 2022 signifies a growing acknowledgment of the significance of research on teacher resilience. As more research is carried out, it becomes crucial to amalgamate and distill the findings, ultimately contributing to a well-rounded comprehension of teacher resilience and its impact on education. Two teacher resilience studies had single authors, while the others included multiple writers. East Asia has the most studies, eleven. Seven European studies followed. Studies covering more than one country were not found in the studies examined. In this context, there is a need to plan studies in which cultural comparisons will be made to examine the resilience levels of teachers from more than one country with multiple variables. Most investigations were effect determination (13), correlation (11), or exploratory (3). Analyzing sample group classification by education level shows that some studies were done at one level and others at numerous levels. Eight primary and seven secondary schools did the most research. The ranking comprised high school (5) and preschool (4). Five studies were undefined, while one was university-level. There is enough studies at the primary and secondary levels. However, studies measuring the resilience levels of university-level lecturers should be planned. EFL instructors worked together when their specializations were examined. Five studies found branch instructors, although two studied preschool teachers and one evaluated special education teachers. In nine inquiries, teacher branches were not explained. Comparisons regarding the specializations of teachers are not dense. It is suggested that the other training that the teachers receive together with their branches should be included in the studies as a variable. It was observed that the preferred scales were mostly nanostructured. It is stated that teachers’ resilience is dynamic and multi-structured. Therefore, it is preferable to use more comprehensive scales. The varying citation counts among the articles underline the wide-ranging influence and impact these studies have had in the teacher resilience research domain. Brouskeli et al. (2018) has the highest citation count, suggesting its notable relevance in the field, while studies like Fernandes et al. (2019) and Liu et al. (2021) also exhibit significant impact. The disparities in citation numbers emphasize the need to consider multiple factors, such as publication date and overarching trends, when assessing the influence of these articles in the field of teacher resilience.

The researchers want to know if certain variables have an impact on the teachers’ assessments of the degree of teacher resilience. First, it was looked at if there were any differences amongst the teachers in terms of demographics like age and gender. Studies show that results vary depending on demographic factors. It is possible to find out whether instructors’ demographic factors interact with other factors (such as being married and having children). Moreover, factors related to instructors’ professions were investigated. For instance, postgraduate teachers are resilient than their undergraduate colleagues. According to study, the impact of teachers’ seniority on their degrees of resilience varies. It is advised to perform research in this area at various educational and cultural levels.

In studies that examine if there is a correlation between teacher resilience and specific variables, the variables are categorized under three headings: psychological variables, variables connected to the workplace, and variables related to teachers’ own competence and qualities. There is a negative correlation between teachers’ resilience and depression, stress, anxiety, well-being, and emotion. On the other hand, dispositional hope, quality of life, well-being, and mattering are positively correlated. In the context of workplace variables, job burnout, and turnover intention are negatively correlated, while job crafting, meaningful work, work engagement, and working conditions are positively correlated. In the context of teachers’ own competence and qualities, resilience was shown to be positively associated with emotion regulation, use of emotion, total emotional intelligence, self-emotional appraisal, empathy, others’ emotion evaluation, teacher competence, teacher self-efficacy, crisis self-efficacy reflection, and self-esteem. In addition to correlation studies, impact studies can also be conducted with these variables, which are thought to be related. Another result is that anger, anxiety, mindfulness, enjoyment, social support, self-efficacy, fear, and training all have an impact on teachers’ resilience. The second group consists of the many factors that are subjected to research about the influence of resilience. Teachers’ resilience influences stress, depersonalization, personal accomplishment, emotional exhaustion, resilience in children, work engagement, happiness, well-being, self-care, and success.

In closing, the increasing research on teacher resilience between 2014 and 2022 highlights its growing significance in education. This mini-review offers a balanced and comprehensive overview of the studies while critically examining their impact. The research landscape is diverse, with varying authorship, locations, and focus. Several variables are associated with teacher resilience, emphasizing the need for further studies exploring resilience levels across different cultural backgrounds, educational levels, and specializations. Additionally, future research should utilize comprehensive, multi-structured scales for a more holistic understanding of teacher resilience. Longitudinal studies can be conducted to determine whether the effects of these variables vary over time. It can also be investigated whether there is a teacher resilience mediating role in the relationship between these variables. The conclusions of this mini-review emphasize the importance of considering multiple factors and taking a critical approach when evaluating research impact in the field of teacher resilience, contributing to the development of effective strategies to support and enhance teacher resilience in various educational contexts.

## Author contributions

All authors listed have made a substantial, direct, and intellectual contribution to the work and approved it for publication.

## Conflict of interest

The authors declare that the research was conducted in the absence of any commercial or financial relationships that could be construed as a potential conflict of interest.

## Publisher’s note

All claims expressed in this article are solely those of the authors and do not necessarily represent those of their affiliated organizations, or those of the publisher, the editors and the reviewers. Any product that may be evaluated in this article, or claim that may be made by its manufacturer, is not guaranteed or endorsed by the publisher.

## References

[ref1] Abdullah R. M. Matore M. E. E. M. Salleh J. M. Adnan R. M. (2019). Relationship between resilience and quality of life (QOL) of special education teachers. Int. J. Innov. Creat. Change 7, 325–335.

[ref2] Ayoobiyan H. Rashidi N. (2021). Can reflective teaching promote resilience among Iranian EFL teachers? A mixed-method design. Reflect. Pract. 22, 293–305. doi: 10.1080/14623943.2021.1873758

[ref3] Baguri E. M. Roslan S. Hassan S. A. Krauss S. E. Zaremohzzabieh Z. (2022). How do self-esteem, dispositional hope, crisis self-efficacy, mattering, and gender differences affect teacher resilience during COVID-19 school closures? Int. J. Environ. Res. Public Health 19, 1–13. doi: 10.3390/ijerph19074150, PMID: 35409829 PMC8998510

[ref4] Benders D. S. Jackson F. A. (2012). Teacher resiliency: nature or nurture? Int. J. Humanit. Soc. Sci. 2, 103–110.

[ref5] Bobek B. L. (2002). Teacher resiliency: a key to career longevity: the clearing house. J. Educ. Strategies Issues Ideas 75, 202–205. doi: 10.1080/00098650209604932

[ref6] Bouillet D. Ivanec T. P. Miljević-Rixički R. (2014). Preschool teachers’ resilience and their readiness for building children’s resilience. Health Educ. 114, 435–450. doi: 10.1108/HE-11-2013-0062

[ref7] Bowles T. Arnup J. L. (2016). Early career teachers’ resilience and positive adaptive change capabilities. Aust. Educ. Res. 43, 147–164. doi: 10.1007/s13384-015-0192-1

[ref8] Brouskeli V. Kaltsi V. Loumakou M. (2018). Resilience and occupational well-being of secondary education teachers in Greece. Issues Educ. Res. 28, 43–60.

[ref9] Campbell-Sills L. Stein M. B. (2007). Psychometric analysis and refinement of the connor–Davidson resilience scale (CD-RISC): validation of a 10-item measure of resilience. J. Trauma. Stress. 20, 1019–1028. doi: 10.1002/jts.20271, PMID: 18157881

[ref10] Cho I. K. Lee J. Kim K. Lee J. Lee S. Yoo S.. (2021). Schoolteachers’ resilience does but self-efficacy does not mediate the influence of stress and anxiety due to the COVID-19 pandemic on depression and subjective well-being. Front. Psych. 12, 1–9. doi: 10.3389/fpsyt.2021.756195 PMC8526840 34690845

[ref11] Connor K. M. Davidson J. R. T. (2003). Development of a new resilience scale: the Connor-Davidson resilience scale (CD-RISC). Depress. Anxiety 18, 76–82. doi: 10.1002/da.10113, PMID: 12964174

[ref12] Daniilidou A. Platsidou M. Gonida S. E. (2020). Primary school teachers’ resilience: association with teacher self-efficacy, burnout and stress. Electron. J. Res. Educ. Psychol. 18, 549–582. doi: 10.25115/ejrep.v18i52.3487

[ref13] Day C. Gu Q. (2014). Response to Margolis, Hodge and Alexandrou: misrepresentations of teacher resilience and hope. J. Educ. Teach. 40, 409–412. doi: 10.1080/02607476.2014.948707

[ref14] Day C. Hong J. (2016). Influences on the capacities for emotional resilience of teachers in schools serving disadvantaged urban communities: challenges of living on the edge. Teach. Teach. Educ. 59, 115–125. doi: 10.1016/j.tate.2016.05.015

[ref15] Diasti K. S. (2021). Constructing professional identity: investigating stress factors and resilience experienced by EFL novice teachers. Scholaria 11, 1–10. doi: 10.24246/j.js.2021.v11.i1.p1-10

[ref16] Ebersöhn L. (2014). Teacher resilience: theorizing resilience and poverty. Teach. Teach. Theory Pract. 20, 568–594. doi: 10.1080/13540602.2014.937960

[ref17] Fernandes L. Peixoto F. Gouveia M. J. Silva J. C. Wosnitza M. (2019). Fostering teachers’ resilience and well-being through professional learning: effects from a training programme. Aust. Educ. Res. 46, 681–698. doi: 10.1007/s13384-019-00344-0, PMID: 36786435

[ref18] Flores M. A. (2020). Preparing teachers to teach in complex settings: opportunities for professional learning and development. Eur. J. Teach. Educ. 43, 297–300. doi: 10.1080/02619768.2020.1771895

[ref19] Gan L. Gao Y. Wu J. (2022). Toward measuring Chinese EFL teachers’ resilience: the role of teachers’ enjoyment, anger, and anxiety. Front. Psychol. 13:853201. doi: 10.3389/fpsyg.2022.853201 35719503 PMC9204088

[ref20] Gratacós G. Mena J. Ciesielkiewicz M. (2021). The complexity thinking approach: beginning teacher resilience and perceived self-efficacy as determining variables in the induction phase. Eur. J. Teach. Educ., 1–18. doi: 10.1080/02619768.2021.1900113

[ref21] Gu Q. Day C. (2013). Challenges to teacher resilience: conditions count. Br. Educ. Res. J. 39, 22–44. doi: 10.1080/01411926.2011.623152

[ref22] Kangas-Dick K. O’Shaughnessy E. (2020). Interventions that promote resilience among teachers: a systematic review of the literature. Int. J. Sch. Educ. Psychol. 8, 131–146. doi: 10.1080/21683603.2020.1734125, PMID: 37016407

[ref23] Khammat A. H. (2022). Investigating the relationships of Iraqi EFL teachers’ emotion regulation, resilience and psychological well-being. Lang. Related Res. 13, 613–640. doi: 10.52547/LRR.13.5.22

[ref24] Kowitarttawatee P. Limphaibool W. (2022). Fostering and sustaining teacher resilience through integration of eastern and Western mindfulness. Cogent Educ 9:2097470. doi: 10.1080/2331186X.2022.2097470

[ref25] Li L. Lv L. (2022). The impact of Chinese EFL teachers’ emotion regulation and resilience on their success. Front. Psychol. 13, 1302–1316. doi: 10.3389/fpsyg.2022.898114 PMC9161025 35664213

[ref26] Liu F. Chen H. Xu J. Wen Y. Fang T. (2021). Exploring the relationships between resilience and turnover intention in Chinese high school teachers: considering the moderating role of job burnout. Int. J. Environ. Res. Public Health 18:6418. doi: 10.3390/ijerph18126418, PMID: 34199322 PMC8296230

[ref27] Liu Y. Zhao L. Su Y. S. (2022). The impact of teacher competence in online teaching on perceived online learning outcomes during the COVID-19 outbreak: a moderated-mediation model of teacher resilience and age. Int. J. Environ. Res. Public Health 19:6282. doi: 10.3390/ijerph19106282, PMID: 35627819 PMC9140542

[ref28] López-Angulo Y. Mella-Norambuena J. Sáez-Delgado F. Peñuelas S. A. P. González O. U. R. (2022). Association between teachers’ resilience and emotional intelligence during the COVID-19 outbreak. Rev. Latinoam. Psicol. 54, 51–59. doi: 10.14349/rlp.2022.v54.6

[ref29] Mackenzie S. (2012). I can’t imagine doing anything else’: why do teachers of children with SEN remain in the profession? Resilience, rewards and realism over time. J. Res. Spec. Educ. Needs 12, 151–161. doi: 10.1111/j.1471-3802.2011.01221.x

[ref30] Mansfield C. F. Beltman S. Broadley T. Weatherby-Fell N. (2016). Building resilience in teacher education: an evidenced informed framework. Teach. Teach. Educ. 54, 77–87. doi: 10.1016/j.tate.2015.11.016

[ref31] Mansfield C. F. Beltman S. Price A. McConney A. (2012). “Don’t sweat the small stuff:” understanding teacher resilience at the chalkface. Teach. Teach. Educ. 28, 357–367. doi: 10.1016/j.tate.2011.11.001

[ref32] Mansfield C. F. Wosnitza M. (2015). Teacher Resilience Questionnaire–Version 1.5. Perth, Aachen: Murdoch University, RWTH Aachen University.

[ref33] Park N. S. Song S. M. Kim J. E. (2020). The mediating effect of childcare teachers’ resilience on the relationship between social support in the workplace and their self-care. Int. J. Environ. Res. Public Health 17, 1–15. doi: 10.3390/ijerph17228513 PMC7698456 33212910

[ref34] Pečjak S. Pirc T. (2022). Teachers’ perceived competence in meeting students’ emotional needs during COVID-19. Psihol. Teme 31, 299–316. doi: 10.31820/pt.31.2.5

[ref35] Peixoto F. Silva J. C. Pipa J. Wosnitza M. Mansfield C. (2020). The multidimensional teachers’ resilience scale: validation for Portuguese teachers. J. Psychoeduc. Assess. 38, 402–408. doi: 10.1177/0734282919836853, PMID: 27409075

[ref36] Smith B. W. Dalen J. Wiggins K. Tooley E. Christopher P. Bernard J. (2008). The brief resilience scale: assessing the ability to bounce back. Int. J. Behav. Med. 15, 194–200. doi: 10.1080/10705500802222972, PMID: 18696313

[ref37] Suryaratri R. D. Yudhistira S. Ulayya D. (2020). The influence of social support towards high school teachers’ resilience in Jakarta, Indonesia, In Proceedings of the 4th International Conference on Learning Innovation and Quality Education

[ref38] Tenorio-Vilchez C. Sucari W. (2021). Understand teacher resilience. A systematic look. Rev. Innov. Educ. 3, 187–197. doi: 10.35622/j.rie.2021.03.012

[ref39] Van Wingerden J. Poell R. F. (2019). Meaningful work and resilience among teachers: the mediating role of work engagement and job crafting. PLoS One 14, 1–13. doi: 10.1371/journal.pone.0222518 PMC6752839 31536544

[ref40] Xie F. (2021). A study on Chinese EFL teachers’ work engagement: the predictability power of emotion regulation and teacher resilience. Front. Psychol. 12, 1–12. doi: 10.3389/fpsyg.2021.735969 PMC8430242 34512487

[ref41] Yirci R. Atalmis E. H. Kiririsci G. (2022). Analyzing the mediating effect of psychological resilience on the relationship between COVID-19 fear and happiness. Educ. Process 11, 147–166. doi: 10.22521/edupij.2022.112.8

